# Research on joint vehicle routing optimization considering multiple distribution centers

**DOI:** 10.1371/journal.pone.0331578

**Published:** 2026-01-23

**Authors:** Di Liu, Mengchi Li, Yushun Lei, Pei Yu, Botang Li, Songyuan Tang

**Affiliations:** 1 School of Sciences and Arts, Guangzhou Maritime University, Guangzhou, China; 2 School of Digital Economics and Trade, Guangzhou Maritime University, Guangzhou, China; 3 School of Maritime Law and Traffic Management, Guangzhou Maritime University, Guangzhou, China; 4 Department of Intelligent Transportation and Engineering, Guangzhou Maritime University, Guangzhou, China; Shanghai Jiao Tong University - Xuhui Campus, CHINA

## Abstract

In order to address the problem of efficiently distributing to multiple demand points within the city and multiple distribution centers on the urban fringes, this paper considers decision-making issues such as the selection of distribution centers and the planning of delivery routes. With the objective of minimizing the total transportation time, an integer programming model for the urban vehicle delivery network is constructed. Moreover, a class of column generation algorithms is designed for the characteristics of the problem. CPLEX is used to compare and analyze the solutions of several examples. The results verify the feasibility of the proposed column generation algorithm, and the practicality of the planning model is demonstrated through the analysis of examples generated from actual data.

## 1. Introduction

The logistics industry, integrating various elements such as transportation, warehousing, and information communication technology, forms a complex composite service network. It serves as a fundamental and strategic industry to support the transformation and upgrading of the manufacturing sector. The rapid growth of the logistics industry is crucial for accelerating the optimization and upgrading of the industrial structure, enhancing the comprehensive service capabilities of the logistics sector, promoting the sustained rapid development of the national economy, and strengthening the overall economic strength of the country [1]. In recent years, with the rise of cross-border e-commerce and the surge in logistics demand, the logistics industry has experienced rapid development and simultaneously faced unprecedented opportunities and challenges for growth. However, at present, most logistics companies have not yet established a scientific and rational logistics distribution information network. Against this backdrop, the design of scientific and reasonable vehicle transportation routes to reduce logistics costs has become a key issue affecting the future development of logistics enterprises.

Currently, most logistics companies employ a decentralized distribution model when executing delivery tasks. This means that the company sets up multiple logistics centers based on actual conditions, with each logistics center handling independent distributions. Delivery personnel arrange vehicle routes based on their personal experience. This distribution model fractures the connections between different distribution centers, preventing the sharing and integration of logistics resources. As a result, some distribution centers may not have vehicles capable of undertaking all transportation tasks, while others may have higher rates of vehicle underutilization. This does not create a comprehensive logistics distribution information network, leading to low efficiency in logistics distribution, a higher proportion of transportation costs, and a range of issues such as low levels of logistics informatization [2], unequal distribution of logistics resources, resource wastage, and low distribution efficiency.

Research on the decentralized distribution model primarily focuses on the construction of optimization models for facility location planning and transportation vehicle routing, and the use of heuristic algorithms for solution [3]. However, the influence of the available multiple distribution centers on facility location and routing optimization is less frequently considered. In real-world urban distribution problems, the selection of distribution centers can affect their deployment locations and numbers. The deployment of distribution centers, in turn, can influence the routing planning of transportation vehicles. The interactive and interdependent decision-making relationships between different optimization objectives make the problem modeling and solution more complex. Therefore, this study considers the joint vehicle routing optimization problem, takes advantage of the structural characteristics of the modeled problem, and designs an efficient exact solution algorithm to integrate logistics resources and improve overall logistics efficiency [4].

In summary, this paper conducts research on the VRP under a multi-center joint distribution model with capacity constraints to meet the enterprise’s delivery requirements considering timeliness, scientifically plan vehicle delivery routes, and improve vehicle delivery efficiency. In addition, this paper develops a column generation algorithm tailored to the model’s characteristics to accommodate the solution of large-scale problems.

The innovations of this paper include: (1) considering multiple distribution centers to increase the diversity of routes, where vehicles can return to another distribution center; (2) more accurately reflecting actual distribution scenarios, making the model closer to reality; (3) designing a column generation algorithm suitable for this problem, this algorithm can accelerate and accurately solve to the optimal solution, improving the efficiency of path generation.

(1) considering multiple distribution centers to increase the diversity of routes; (2) more accurately reflecting actual distribution scenarios, making the model closer to reality; (3) designing a column generation algorithm suitable for this problem.

The rest of this paper is organized as follows. Section 2 introduces related work. Section 3 provides background on the problem and describes the composition of the model. Section 4 describes the algorithm. Section 5 presents numerical experiments, and Section 6 summarizes the results.

## 2. Literature review

The Vehicle Routing Problem (VRP) has received extensive attention from scholars both domestically and abroad. Currently, scholars have conducted extensive research on the related models and solution algorithms for the vehicle routing problem. Therefore, the following sections will provide a detailed explanation of the multi-center vehicle routing problem and the solution algorithms for the vehicle routing problem.

### 2.1. Classic vehicle routing problem

Since its inception, the VRP has been widely researched, with scholars applying the theory to practical issues. Jolfaei and Alinaghian [5] developed a VRP model with roaming delivery locations and hard time windows, aiming to minimize total travel time. Xiao et al. [6] proposed an extended low-carbon VRP model considering dynamic speed, steep slopes, and load impacts on emissions, alongside an adaptive large neighborhood search algorithm, verified via numerical examples, and derived management insights. Masmoudi et al. [7] studied a new multi-room waste collection VRP, incorporating separation and refilling points, and developed a mixed-integer linear programming model with bin washers, demonstrating efficient solutions via numerical examples. Cavaliere et al. [8] specialized the FILO framework for capacity-constrained VRPs to address simultaneous pickup and delivery, and mixed pickup and delivery challenges. Alvarez et al. [9] addressed a VRP with random customer and demand consistency using a two-phase random programming framework, branch-and-cut, and Benders decomposition, proposing a sample average approximation method. Kou et al. [10] applied linear regression models to estimate optimal solution values for divergent distribution VRPs, proposing a model integrating topological features and solution value statistics.

### 2.2. Algorithm for solving vehicle routing problems

Scholars have developed heuristic algorithms for the VRP. Parada et al. [11] introduced an integer L-shaped method for two-phase stochastic integer programming, utilizing new optimality cuts and lower bounds. Rodríguez-Esparza et al. [12] proposed a hyperheuristic method, the Hyperheuristic Adaptive Simulated Annealing Reinforcement Learning Algorithm, for the capacitated electric VRP. Ou et al. [13] suggested a constrained evolutionary optimization algorithm for a multi-objective VRP with simultaneous delivery, pickup, and time windows. Wang et al. [14] studied the impact of increased package delivery volume on city accessibility and livability, presenting a multi-tiered distribution system model with multiple transportation modes and time frames, and introduced a stochastic utility discrete choice model. An adaptive large neighborhood search algorithm was developed. Su et al. [15] constructed a green VRP with time windows and multiple parking lots, aiming to optimize warehouse quantity and location using a lightweight genetic algorithm with variable neighborhood search and an enhanced crossover operator. Fragkogios et al. [16] focused on the last-mile urban cargo delivery VRP, considering multiple vehicle trips, time-varying travel times, customer time windows, and loading times. A simplified model was proposed using Benders method for subproblem decomposition without a duality gap.

Another group of scholars use precise algorithms to solve problems, such as Glize et al. [17] embedding an effective single objective algorithm into constraint methods. The resulting single objective optimization problem can be solved using state-of-the-art methods based on column generation, and mechanisms and techniques to accelerate the speed of the generated algorithm have been proposed. Wu et al. [18] solved a split pickup vehicle routing problem with time windows and time-dependent demands, where different customer demands are generated at different constant rates and vehicles are allowed to pick up goods during the demand generation process. This problem is expressed as a mixed integer nonlinear programming model, and a sequence extension network is designed to represent the relationships between multiple pickups. A branch pricing segmentation algorithm based on nested column generation is proposed to solve this problem. Duman et al. [19] focused on the electric vehicle routing problem with spatiotemporal constraints and developed a novel column generation method embedded in a bidirectional branch pricing algorithm, enhanced the pulse program, and adopted an enhanced bidirectional algorithm to solve the pricing subproblem, overcoming common weaknesses of classical labeling algorithms such as label storage. Huang et al. [20] investigated a multi travel vehicle routing problem involving downgraded multi skilled human resources, with the goal of finding the optimal vehicle routing plan and multi skilled human resource scheduling plan while minimizing total costs (including travel and employee costs) without violating time windows and lunch break restrictions. For this purpose, we have developed two mathematical models: an arc flow model and a set coverage model based on travel. In addition, a branch pricing segmentation algorithm based on the set coverage model was proposed to solve practical scale instances. However, the algorithms mentioned above are difficult to apply to the background and model of this article.

### 2.3. Variants of vehicle routing problem

Some researchers have studied variants of the VRP. Lehmann and Winkenbach [21] introduced a new, extensive two-tier VRP variant that combines important real-world features such as time windows, mixed pickup and delivery requirements, vehicle range limitations, and multiple trips per second-tier vehicle. They proposed a compact mathematical model that solves small examples optimally within a reasonable time and an adaptive large neighborhood search algorithm that integrates the precise formulas of the first-tier route into the second-tier route to solve medium and large instances. Rist et al. [22] studied a new heuristic method for exact solution of inequalities based on Benders’ decomposition for the active-passive VRP, which involves complex time synchronization requirements among vehicles. Huang and Wang [23] investigated the optimization of large-scale, dense offshore wind farms with total detection energy consumption, using mobile edge computing to assist in automatic drone detection. They built a combinatorial optimization model based on the VRP with distance constraints and capacity limits and proposed an improved hybrid heuristic algorithm based on K-means clustering, minimum enclosing circle, and the Lin-Kenighan-Helsgaun algorithm to find the optimal locations and quantities of automatic drone airports. Ferreira et al. [24] solved the green VRP with two-dimensional loading constraints and split deliveries, proposing the first metaheuristic method based on variable neighborhood search and designing effective routes using various strategies (like lower bound procedures, open space heuristics, and constraint programming models) to ensure the feasibility of loading constraints. Zhou et al. [25] developed a mixed-integer linear programming model for a new VRP variant, the two-tier time-varying VRP with simultaneous pickup and delivery and satellite synchronization, where customers are divided into multiple customer regions based on different geographic features and proposed a memetic algorithm that includes a split algorithm for chromosome decoding and a three-stage constructive heuristic algorithm for initial population generation. However, the above papers use heuristic ideas and column generation algorithms less frequently and solve large-scale problems using small-scale models.

### 2.4. Vehicle routing problem for multi center joint distribution

Multi-center joint distribution involves coordinating two or more distribution centers to service multiple customer points through unified dispatch. This approach addresses challenges in sharing logistics resources and minimizing inefficiencies in vehicle routing. Research includes: Rahmanifar et al. [26] on waste collection logistics with a two-tier routing model and heuristic algorithms; Souza et al. [27] on electric vehicle and battery swapping station routing; Wu et al. [18] on pickup truck routing with time windows and time-dependent demands; Li et al. [28] on dynamic vehicle routing with capacity constraints; Zhang et al. [29] on collaborative vehicle routing with simultaneous pickup and delivery; and Wang et al. [30] on smart recycling bin routing and pricing with resource sharing. However, the issue of vehicles at city edges not needing to return to the original distribution center is largely unexplored.

Table 1 summarizes the characteristics of the main references and the features of this article. The contributions of existing literature in Table 1 include: (1) considering many variants of vehicle routing problems; (2) Use heuristic algorithms and exact algorithms separately or simultaneously to solve the proposed problem.

**Table 1:** Summary of literature review.

No.	Paper	Multiple distribution centers	Joint vehicle	Uncertainty	Heuristic algorithm	Benders’ decomposition	Branch and bound method	Column generation
1	Jolfaei and Alinaghian [5]	No	No	Yes	No	No	No	No
2	Cavaliere et al. [8]	No	No	Yes	Yes	No	No	No
3	Kou et al. [10]	No	No	No	Yes	No	No	No
4	Rodríguez-Esparza et al. [12]	No	No	No	Yes	No	No	No
5	Su et al. [15]	No	No	No	Yes	No	No	No
6	Glize et al. [17]	No	No	No	No	No	No	Yes
7	Wu et al. [18]	No	No	Yes	No	No	Yes	No
8	Duman et al. [19]	No	No	No	No	No	Yes	No
9	Huang et al. [20]	No	No	No	No	No	Yes	No
10	Rist et al. [22]	No	No	No	No	No	No	No
11	Ferreira et al. [24]	No	No	Yes		Yes	No	No
12	Rahmanifar et al. [26]	Yes	No	No	Yes	No	No	No
13	Wu et al. [18]	Yes	No	Yes		No	No	No
14	Zhang et al. [29]	Yes	No	No		No	No	No
15	This research	Yes	Yes	No	No	No	No	Yes

In summary, joint vehicle routing is a relatively new topic, although many researchers have been studying it. However, there is a lack of research on VRPs from the perspective of vehicles not needing to return to the original distribution center. Therefore, this paper aims to optimize joint vehicle routing paths from the perspective of vehicles not needing to return to the original distribution center.

## 3. Problem description and modeling

### 3.1. Problem description

Given the high cost of urban land, many distribution centers are constructed in the city’s suburbs or on its fringes. This results in scenarios where vehicles begin from one corner of the city, deliver to various demand points within it, and then proceed to a different corner to a distribution center. This issue can be conceptualized as a joint vehicle routing optimization problem involving multiple distribution centers. This problem entails planning the routes of vehicles that originate from several distribution centers to provide logistics services to customers located throughout the city. In this model, vehicles and resources are shared across different distribution centers, which removes the previous limitation that each customer point could only be served by a single distribution center. In addition, the origin of the vehicle from which distribution center should be determined based on the last distribution center where the vehicle stayed in the previous cycle. Furthermore, vehicles are not returning to the original distribution center from which they set out; they now have the flexibility to return to different centers. Consequently, this paper aims to optimize the routing of these vehicles while ensuring that the weight constraints of the vehicles are met, with the ultimate goal of minimizing the total transportation time.

**Fig 1:**
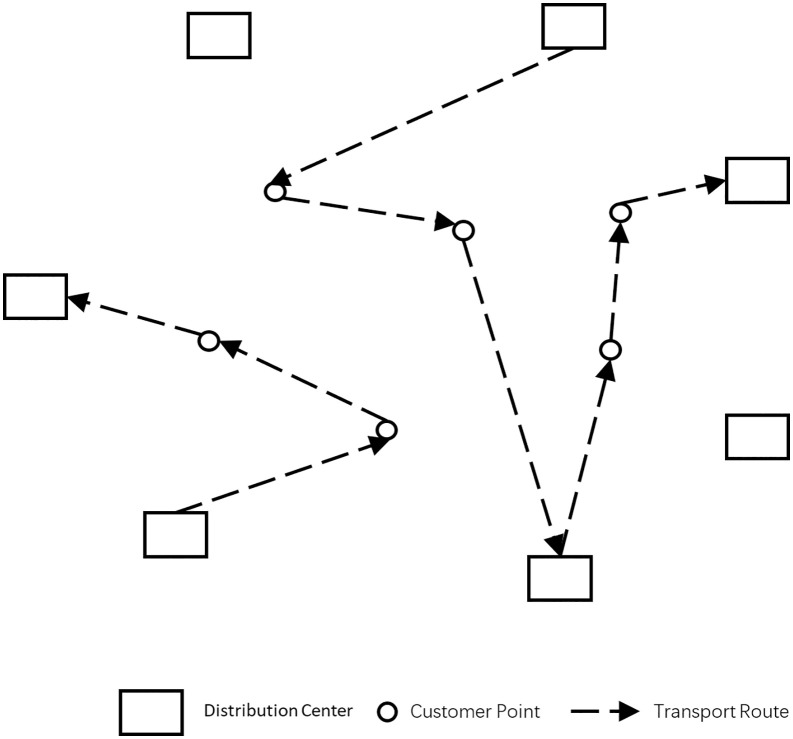
Schematic of the multi-center joint distribution model.

Here is a detailed description of the vehicle distribution process: Multiple distribution centers jointly provide distribution services to customer points, with vehicles setting out from different centers to consecutively serve each customer point. If a vehicle is empty or if the last customer point on its route has a demand greater than the vehicle’s remaining carrying capacity, the vehicle may return to another distribution center. This process is repeated until all customer points have been served. The joint distribution method enables distribution centers to expand their service areas, breaking geographical constraints between customer points. Furthermore, when a vehicle is empty or unable to serve the next customer point due to insufficient load, it can choose to return to the nearest distribution center. Additionally, in cases where a distribution center is out of stock and unable to meet the needs of its assigned customer points, the joint distribution model allows other centers to step in and fulfill the demands of the customer points. The schematic diagram of the multi-center joint distribution model is illustrated in **Fig 1**.

### 3.2. The model

#### 3.2.1. Assumptions.

To facilitate the solution of the model, the following assumptions are made in the model established in this paper:

(1) All vehicles must start from the distribution center, deliver goods to designated customers, and then return to the distribution center, or return to other distribution centers;
(2) The locations of customers and distribution centers are fixed and known;
(3) The demand of all customer points is known, and the transport capacity of each vehicle is sufficient to meet the demand of a single customer. Each customer point is served by only one vehicle and can only be served once.;
(4) The demand of all customer points is known and needs to be satisfied. Each customer point is served by only one vehicle and can only be served once;
(5) The load capacity of the vehicles cannot exceed their maximum rated load capacity.

#### 3.2.2. Symbol explanation.

The parameters and variables used in this model are defined as follows:

(1) Set

**Table:** 

I	City customer point set, I={1,2,⋯,NI}, where NI is the total number of customer points in the city;
J	The collection of customer points within the city and distribution centers at the city’s edge, where J={0,1,2,⋯,NI}, and 0 represents the distribution center at the city’s edge;
K	Vehicle set, where K={1,2,⋯,NK}, and NK represents the total number of vehicles;
L	Peripheral distribution center set, where L={1,2,⋯,NL}, and NL represents the total number of peripheral distribution centers.

(2) Parameters

**Table:** 

$t_{ij}$	The average walking time between customer points i and j within the city, i,j∈I, min;
$T_{li}$	The average walking time between the peripheral distribution center l and the city customer point i, l∈L,i∈I, min;
M	An adequately large value, This parameter is an unitless rational number greater than Q;
$d_{i}$	The demand of customer point i within the city;
Q	The loading capacity of the transportation vehicles within the city, t.

(3) Decision variables

**Table:** 

$x_{kij}$	0-1 variable, which is 1 when vehicle k passes through the city customer point i and then through j, otherwise 0, k∈K,i,j∈J;
$u_{ki}$	The cumulative total distribution volume of vehicle k after passing through the city customer point i, k∈K,i∈J;
$y_{kli}$	0-1 variable, which is 1 when vehicle k travels from the peripheral distribution center l to the first city customer point i, otherwise 0, k∈K,l∈L,i∈I;
${y^{\prime }}_{kli}$	0-1 variable, which is 1 when vehicle k travels from the last city customer point i to the peripheral distribution center l, otherwise 0, k∈K,l∈L,i∈I.

#### 3.2.3 Programming model.

Based on the above description, a mathematical model is established for the joint vehicle routing optimization problem considering multiple distribution centers. The objective function is to minimize the total walking time, which includes the time for vehicles to travel from distribution centers to the first customer points, the transit time between two customer points, and the time for the last customer point to return to the distribution center, among others.


$$\text{min}\sum_{l\in L}\sum_{k\in K}\sum_{i\in I}T_{li}y_{kli}+\sum_{k\in K}\sum_{i\in I}\sum_{j\in I}t_{ij}x_{kij}+\sum_{k\in K}\sum_{l\in L}\sum_{i\in I}T_{li}{y^{\prime }}_{kli}$$ (1)

According to the problem description, the model established in this paper must satisfy the following constraints:


$$\sum_{k\in K}\sum_{i\in J}x_{kij}=\text{1},\;\;\forall j\in I,i\neq j$$ (2)

$$\sum_{j\in I}x_{k\text{0}j}\leq \text{1},\;\;\forall k\in K$$ (3)

$$\sum_{i\in J}x_{kij}=\sum_{i\in J}x_{kji},\;\;\forall k\in K,j\in I$$ (4)

$$\sum_{i\in I}x_{ki\text{0}}\leq \text{1},\;\;\forall k\in K$$ (5)

$$\sum_{l\in L}y_{klj}\geq x_{k\text{0}j},\;\;\forall k\in K,j\in I$$ (6)

$$\sum_{l\in L}{y^{\prime }}_{kli}\geq x_{ki\text{0}},\;\;\forall k\in K,i\in I$$ (7)

$$\sum_{i\in I}y_{kli}+\sum_{i\in I}{y^{\prime }}_{kli}\leq \text{1},\;\;\forall k\in K,l\in L$$ (8)

$$u_{ki}+d_{i}x_{kij}-M\left(\text{1}-x_{kij}\right)\leq u_{kj},\;\;\forall k\in K,i,j\in I$$ (9)

$$\sum_{i\in I}\sum_{j\in J}d_{i}x_{kij}\leq Q,\;\;\forall k\in K$$ (10)

$$x_{kij},y_{kli},{y^{\prime }}_{kli}\in \left\{\text{0},\text{1}\right\},\;\;\forall k\in K,l\in L,i,j\in J$$ (11)

$$u_{ki}\geq \text{0},\;\;\forall k\in K,i\in J$$ (12)

Among them, equation (2) ensures that each customer point is visited only once. Equation (3) stipulates that if a vehicle visits a customer point, it must have originated from a distribution center. Equation (4) represents the conservation of flow, meaning that vehicles entering a customer point for service must also leave from that customer point. Equation (5) specifies that if a route visits a customer point, it must return to a certain distribution center. Equations (6) and (7) limit that vehicles with delivery tasks must select a starting and ending distribution center for their route. Equation (8) restricts that the starting and ending distribution centers for the same route cannot be the same. Equation (9) is used to accumulate the total goods transported along the route, the function of this inequality is to accumulate the number of demand points that the line passes through for points that are connected, while for points that are not connected or have no relationship, the cumulative demand of the two points is not directly related; equation (10) limits the total transported volume along the route to the capacity of the transportation vehicles within the city. Equation (11) restricts the variables to binary variables (0 or 1). Equation (12) restricts the variables to integers.

The above model contains a large number of constraints and variables, and it grows rapidly with the increase in the number of customer points. Even for small-scale numerical examples, it is difficult to find a high-quality solution within a limited time. Therefore, based on the characteristics of the model, an exact solution algorithm based on column generation is designed.

## 4 Solution method

Column generation is an improvement over the simplex method, where in the iterative process of determining entering and leaving base variables, only one variable is involved in each iteration. This means that only a small portion of variables are involved in the entire solution process. By proposing the column generation algorithm, each target is transformed into finding a non-base variable with a negative test number to enter the base, which can greatly improve the efficiency of the solution.

The vehicle routing optimization problem with multiple distribution centers considered in this paper is a multi-vehicle planning problem with capacity constraints. By referring to Gaura and Singh [31], the above model is decomposed into a master problem based on paths and a subproblem of shortest paths with resource constraints.

### 4.1 Column generation algorithm without vehicle quantity constraint (OQCGA)

#### 4.1.1 Master problem.

The master problem model constructed in this paper is a 0–1 planning model for the path matrix, as shown by equations (13) to (15). The set of paths is denoted as P, where p∈P, representing the pth path executed by the vehicle. The variable $\alpha_{p}$ represents the minimum accumulated time required to satisfy the customer demand included in path p. For each path p, when passing through customer i, $\omega_{ip}=\text{1}$; otherwise, $\omega_{ip}=\text{0}$. The variable $\beta_{p}$ is a 0–1 decision variable that indicates whether to choose path p; if selected, then $\beta_{p}=\text{1}$, otherwise $\beta_{p}=\text{0}$.


$$\text{min}\sum_{p\in P}\alpha_{p}\beta_{p}$$ (13)

$$\text{s}.\text{t}.\;\sum_{p\in P}\omega_{ip}\beta_{p}=\text{1},\;\;\forall i\in I$$ (14)

$$\beta_{p}\in \left\{\text{0},\text{1}\right\},\;\;\forall p\in P$$ (15)

Equation (13) represents the minimization of the total path travel time; equation (14) indicates that each customer must be visited. By combining the subproblem, new paths can be continuously generated and added to the set P.

#### 4.1.2 Subproblem.

Let $\theta_{i}$ be the dual variable of equation (14), and the dual problem corresponding to the main problem is:


$$\text{max}\sum_{i\in I}\theta_{i}$$ (16)

$$\text{s}.\text{t}.\;\sum_{i\in I}\omega_{ip}\theta_{i}\leq \alpha_{p},\;\;\forall p\in P$$ (17)

$$\theta_{i}\;\text{is}\;\text{unconstrained},\;\;\forall i\in I$$ (18)

Combining equations (1) to (12), the corresponding subproblem model is as follows


$$\text{min}\sum_{l\in L}\sum_{i\in I}T_{li}y_{li}+\sum_{k\in K}\sum_{i\in I}\sum_{j\in I}t_{ij}x_{ij}+\sum_{l\in L}\sum_{i\in I}T_{li}{y^{\prime }}_{li}-\sum_{i\in I}\left(\theta_{i}\sum_{j\in J}x_{ji}\right)$$ (19)

$$\text{s}.\text{t}.\;\sum_{j\in I}x_{\text{0}j}=\text{1}$$ (20)

$$\sum_{i\in I}x_{i\text{0}}=\text{1}$$ (21)

$$\sum_{i\in J}x_{ij}\leq \text{1},\;\;\forall j\in I$$ (22)

$$\sum_{i\in J}x_{ij}=\sum_{i\in J}x_{ji},\;\;\forall j\in I$$ (23)

$$\sum_{l\in L}y_{lj}\geq x_{\text{0}j},\;\;\forall j\in I$$ (24)

$$\sum_{l\in L}{y^{\prime }}_{li}\geq x_{i\text{0}},\;\;\forall i\in I$$ (25)

$$\sum_{i\in I}y_{li}+\sum_{i\in I}{y^{\prime }}_{li}\leq \text{1},\;\;\forall l\in L$$ (26)

$$u_{i}+d_{i}x_{ij}-M\left(\text{1}-x_{ij}\right)\leq u_{j},\;\;\forall i,j\in I$$ (27)

$$\sum_{i\in I}\sum_{j\in J}d_{i}x_{ij}\leq Q$$ (28)

$$x_{ij},y_{li},{y^{\prime }}_{li}\in \left\{\text{0},\text{1}\right\},\;\;\forall l\in L,i,j\in J$$ (29)

$$u_{i}\geq \text{0},\;\;\forall i\in J$$ (30)

The meaning of this model is to find the path with the smallest test number under the optimal solution of the current master problem, which can be seen as a shortest path solving problem. The solutions with test numbers less than zero are taken as new columns and inserted into the master problem for iteration to optimize the objective function value of the master problem.

### 4.2 Column generation algorithm with vehicle quantity constraint (QGCA)

#### 4.2.1 Master problem.

The master problem model mentioned in section 4.1, i.e., equations (13) to (15), does not consider the issue of vehicle quantity constraints. Based on this, the main model is rephrased as follows with the constraint on vehicle quantity:


$$\text{min}\sum_{p\in P}\alpha_{p}\beta_{p}$$ (31)

s.t. equation (14), (15)


$$\sum_{p\in P}\beta_{p}\leq NK$$ (32)

Among them, the meaning of *NK* is consistent with section 3.2.2, indicating the number of vehicles owned.

#### 4.2.2 Subproblem.

Let $\theta_{i}$ and μ be the dual variables of equations (14) and (32), respectively. The dual problem corresponding to the master problem is:


$$\text{max}\sum_{i\in I}\theta_{i}-NK\times \mu$$ (33)

$$\text{s}.\text{t}.\;\sum_{i\in I}\omega_{ip}\theta_{i}-\mu \leq \alpha_{p},\;\;\forall p\in P$$ (34)

$$\theta_{i}\;\text{is}\;\text{unconstrained},\mu \geq \text{0},\;\;\forall i\in I$$ (35)

The corresponding subproblem model is as follows


$$\text{min}\sum_{l\in L}\sum_{i\in I}T_{li}y_{li}+\sum_{k\in K}\sum_{i\in I}\sum_{j\in I}t_{ij}x_{ij}+\sum_{l\in L}\sum_{i\in I}T_{li}{y^{\prime }}_{li}-\sum_{i\in I}\left(\theta_{i}\sum_{j\in J}x_{ji}\right)+\mu$$ (36)

s.t. equations (20) to (30)

In summary, since OQCGA did not consider the actual transportation process where there is a limit on the number of vehicles, QGCA was added to make the model more realistic. Although OQCGA can select routes with actual transportation based on the results obtained, it will add many useless routes that occupy computer memory. Therefore, increasing the limit on the number of vehicles can reduce the generation of unnecessary sub routes. Meanwhile, this article proposes two algorithms to observe the changes in solution time and target values after reducing or increasing constraint conditions.

### 4.3 Steps of the column generation algorithm

Using column generation, we compute a feasible solution to the linear programming relaxation of the master problem. Each column of $\omega_{ip}$ represents a feasible route along with its associated cost $\alpha_{p}$. The order in which customers are visited within the route is notprescribed in the master problem but can be determined in the subproblem. We assume that customers are visited sequentially and that the route forms a loop with the minimum accumulated cost. Calculating the minimum cost $\alpha_{p}$ also yields the optimal visit order. The column generation algorithm begins with a set of initial feasible columns, known as individual matrices. These represent a route that starts at the origin, visits a set of customer points, and returns to the origin. The linear relaxation of the master problem is then solved to identify cost reductions from the current feasible solution, and the dual variables are applied to the subproblem to generate a new column with a reduced cost. The subproblem is solved exactly using CPLEX’s dynamic programming solver, which incorporates columns that reduce costs into the current feasible solution. If no such columns are generated in the subproblem, the algorithm terminates. Algorithm 1 provides a pseudocode representation of the two column generation algorithms described above. Among them, the meanings of NI,NJ,NK, and all related symbols in this section are consistent with those in section 3.2.2.

**Algorithm 1:** Pseudocode for the Column Generation Algorithm

Step 1: Input parameters $NI,NJ,NK,NL,t_{ij},T_{li},M,d_{i},Q$;

Step 2: Let $R_{ij}$ be an NI-dimensional unit matrix, i,j∈I, $\alpha_{p}$ be an NI-dimensional zero vector, p∈I;

Step 3: Let $\omega_{ip}=R_{ij}$;

Step 4: Let $\alpha_{p}=\text{min}\left(T_{lp}\right)+\left(the\;second\;smallest\;value\;of{\;T}_{lp}\right)$;

Step 5: Solve the relaxed master problem to find the optimal dual variable $\theta_{i},\mu$;

Step 6: Call CPLEX’s dynamic programming optimization method with the dual variable $\theta_{i},\mu$ as input to solve the subproblem;

Step 7: Obtain the minimum cost path, a vector q of length NI, and its cost C(q);

Step 8: **while** C(q)<0;

Step 9: Add column q to $\omega_{ip}$ and C(q) to $\alpha_{p}$;

Step 10: Solve the relaxed master problem to find the optimal dual variable $\theta_{i},\mu$;

Step 11: Call CPLEX’s dynamic programming optimization method to solve the subproblem;

Step 12: Obtain the minimum cost path, a vector q of length NI, and its cost C(q);

Step 13: **end**;

Step 14: Derive the objective value and its solution from the master-sub problem at the final step.

Algorithm 1 proceeds as follows: In steps 1–4, the master problem is initialized with an identity matrix. This initial route begins at the distribution center closest to the customer, visiting a single customer, and then returning to the second closest distribution center. In step 5, the relaxed master problem is solved, and the optimal dual variables are calculated based on the given initial basis. Steps 6 and 7 involve utilizing CPLEX’s dynamic programming optimization method, leveraging the dual variables to identify the path with the greatest cost reduction. If CPLEX’s dynamic programming yields a column representing a cost reduction, the process is iterated. If not, the while loop is terminated. The algorithm concludes by extracting the objective value and its corresponding solution from the master-sub problem at the final step.

## 5 Numerical experiments

### 5.1 Input data generation

To verify the efficacy of the model and algorithm, this study employs data from a department store within Guangzhou, China to populate the model for resolution and analysis. Furthermore, a set of diverse numerical examples was created by randomly sampling within the reasonable numerical bounds of the survey data to evaluate the algorithm’s efficiency. The model, presented in equations (1) through (2), was coded and solved using CPLEX 12.8 software. The two column generation algorithms proposed were implemented in MATLAB 2015b, augmented by the YALMIP toolbox, and leveraged the CPLEX 12.8 algorithm package for solution processing. These applications were conducted on a portable computer featuring an Intel(R) Core(TM) i7-7500U CPU @ 2.70GHz, operating at 2.90 GHz. Drawing from the real-world survey data, the paper provides the scope of the relevant data as shown in Table 2, and generated 30 numerical examples of varying sizes based on the data in Table 2 (see Table 3), with Q set to reflect the carrying capacity of a 9.6-meter truck, which is 18 tons.

**Table 2:** Relevant data.

Parameters	Range
$t_{ij}$	U(5,120)min
$T_{li}$	U(5,150)min
$d_{i}$	U(2,10)t
Q	18t

**Table 3:** Example size.

Numerical examples	Number of distribution centers	Number of stores	Number of vehicles
1	10	10	3
2	11	12	4
3	11	15	5
4	12	17	6
5	12	20	7
6	13	22	8
7	13	25	9
8	14	27	10
9	14	30	11
10	15	32	12
11	15	35	13
12	16	37	14
13	16	40	15
14	17	42	16
15	17	45	17
16	18	47	18
17	18	50	19
18	19	52	20
19	19	55	21
20	20	57	22
21	21	60	23
22	22	62	24
23	23	65	25
24	24	67	25
25	25	70	25
26	26	72	26
27	27	75	27
28	28	77	28
29	29	78	29
30	30	80	30

### 5.2. Evaluation of the candidate solution approaches

Using CPLEX, OQCGA, and QGCA algorithms to solve 30 numerical examples, Table 4 was produced. Among the 30 examples of varying sizes, the two column generation algorithms achieved function values identical to the exact solutions found by CPLEX. When the problem size increased to example 19, CPLEX’s solving time exceeded one hour, and it could only find feasible solutions, whereas both column generation algorithms could still find relatively good solutions within a relatively short time. When the problem size expanded to example 23, CPLEX had to resort to heuristic algorithms, while both column generation algorithms could still produce near-optimal solutions within an acceptable time frame. This demonstrates that the two column generation algorithms proposed in this paper exhibit superior performance in terms of shorter solving time and relatively better solution quality for the joint vehicle routing problem with multiple distribution centers. For the OQCGA and QGCA algorithms, although OQCGA generally has a shorter solving time than QGCA, QGCA achieves the smallest target values.

**Table 4:** Comparison of CPLEX with OQCGA and QGCA algorithm results.

Numerical examples	CPLEX		OQCGA		QGCA	
	Optimal solution	Solving time	Objective value	Solving time	Objective value	Solving time
1	**87**	6.73	87	0.91	87	1.27
2	**91**	7.56	91	15.64	91	18.36
3	**95**	7.46	95	23.85	95	25.74
4	**98**	9.86	98	19.35	98	26.84
5	**102**	10.86	102	56.78	102	63.74
6	**106**	20.84	106	31.06	106	26.98
7	**109**	35.49	109	136.28	109	159.86
8	**115**	63.89	115	149.30	115	183.02
9	**118**	1314.68	118	185.37	118	216.85
10	**121**	2935.47	121	165.63	121	221.30
11	**126**	3248.09	126	286.25	126	296.78
12	**129**	4632.99	129	371.54	129	398.46
13	**132**	5836.27	132	396.16	132	423.89
14	**135**	6893.28	135	427.35	135	463.89
15	**139**	7859.42	139	572.89	139	634.29
16	**141**	8638.29	141	837.69	141	932.78
17	**158**	8963.75	158	947.52	158	986.34
18	165	9688.20	163	1092.84	161	1163.52
19	173	>9600	171	1357.45	168	1587.03
20	186	>9600	182	1687.59	179	1874.26
21	196	>9600	193	1967.80	188	2136.54
22	213	>9600	208	2637.63	202	2863.83
23	--	employ heuristic algorithms	235	3077.54	215	3114.85
24	--	--	296	2968.12	263	3068.09
25	--	--	317	3209.03	289	3275.96
26	--	--	332	3549.83	320	3623.09
27	--	--	362	3875.19	358	3784.28
28	--	--	395	3965.27	386	4029.38
29	--	--	415	4567.16	412	4621.95
30	--	--	469	4857.06	466	4819.63

There are best, average, and worst case scenarios for the time complexity of algorithms. The worst-case scenario refers to the maximum number of runs (upper bound) for any input size. The average situation is the expected number of runs for any input size. The best case scenario is the minimum number of runs (lower bound) for any input size. And the focus of this article is on the best case scenario of the algorithm. In this article, due to the special solving mechanism of CPLEX, the final solution can only be obtained after the solution is completed. The OQGCA algorithm can only obtain a convergence value after more than two-thirds of the total running times, while the QCGA algorithm proposed in this paper can stably obtain a convergence value before half of the total running times. Therefore, it indicates that the QCGA algorithm is relatively stable.

### 5.3. Sensitivity analysis and managerial insights

**Fig 2:**
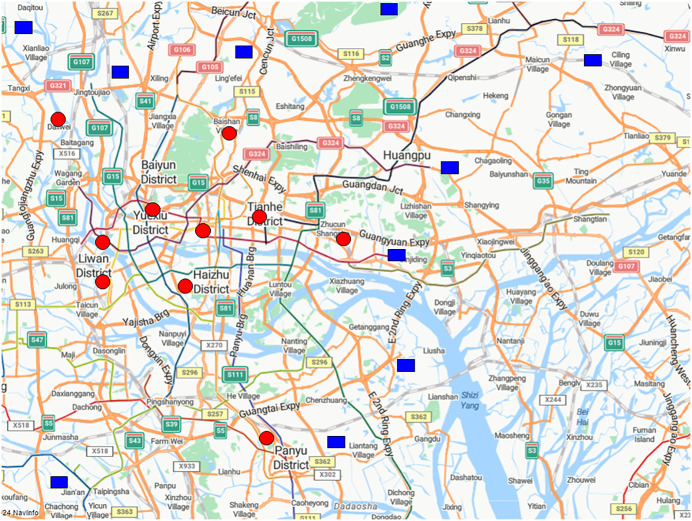
Map of distribution centers locations.

To analyze the impact of multiple distribution centers on the joint vehicle routing problem, this study conducts an analytical case study. J Supermarket, a well-known domestic enterprise, has hundreds of storefronts in the Guangzhou area. Due to the concentration of stores in the city center area of Guangzhou, this paper chooses the customer storefronts and related distribution center data in this region. As shown in Fig 2, through research and data investigation, the enterprise has 10 customer storefronts (red circles) and 10 distribution centers (blue rectangles) in the central area of Guangzhou, and sets 3 vehicles. The travel time matrix between the two types of nodes and between them can be obtained through the driving mode navigation of Microsoft Bing Maps. The demand of customers is respectively taken as 5t, 4t, 5t, 3t, 6t, 5t, 3t, 2t, 6t, and 3t. The optimal value of 87 is obtained using the QGCA algorithm, and the results of Table 4 (the parentheses in the table represent the selected distribution centers) are obtained.

As shown in Table 5, in practical applications, by employing the method proposed in this paper, the enterprise can optimize the daily delivery solutions for retail businesses based on the objective of time efficiency for that week. Overall, the optimization model proposed in this paper is aligned with real-world scenarios, and the decisions provided reflect practicality and effectiveness. Additionally, the model offers decision-making support for business managers.

**Table 5:** Optimal routes.

Vehicles	Routes
1	(10)0→4→7→8→3→6→0(3)
2	(1)0→5→0(6)
3	(9)0→9→1→10→2→0(2)

## 6. Conclusion

With the development of the economy and the rise of cross-border e-commerce, the logistics industry has shown a robust growth momentum. Due to the characteristics of economy, uncertainty, and multi-objective nature in the logistics distribution process, higher requirements are placed on vehicle routing planning. In the process of vehicle delivery, how to meet customer demands while completing the delivery task at a lower cost has become a primary issue in current research. This paper starts from the perspective of multiple distribution centers, comprehensively considering the factors affecting vehicle load, and constructs a mathematical model with the objective function of minimizing the total time. The research focuses on the multi-center joint vehicle routing problem and utilizes two column generation algorithms to solve it.

As the economy grows and cross-border e-commerce emerges, the logistics industry is experiencing a period of vigorous expansion [32,33]. The distribution process in logistics presents economic, uncertain, and multi-objective traits, which demand more stringent criteria for vehicle routing planning. The challenge lies in achieving customer satisfaction while minimizing costs during vehicle deliveries. This paper takes a multi-distribution center approach, integrating factors such as vehicle load capacity, and develops a mathematical model with the objective of minimizing the total delivery time. The study addresses the complexities of the multi-center joint vehicle routing problem and employs two column generation algorithms to arrive at optimal solutions.

In addressing the multi-center vehicle routing problem with carbon emissions considerations, the independent distribution model often leads to issues such as low resource utilization and higher costs. Recognizing that collaboration among multiple centers can enable resource sharing, this paper proposes a multi-center joint distribution strategy, which extends the research scope of distribution centers and vehicle routing problems, truly achieving “many-to-many” overall planning. Furthermore, by selecting example data from Guangzhou, two column generation algorithms were designed to enhance the search capabilities, enabling the discovery of better results within the solution space. In considering the multi-center joint vehicle routing problem, this paper assumes all factors to be deterministic and does not account for uncertain variables. However, in the real-world distribution process, uncertainties such as traffic congestion, area restrictions, and weather changes are inevitable. Therefore, future research should focus on vehicle routing under uncertain conditions. Building on this, the algorithm could be combined with other emerging algorithms or theories (such as the Markov decision process, dynamic programming), or more detailed operational operators could be introduced to improve the precision of the algorithmic solutions.

**Institutional Review Board Statement**: This article does not contain any studies with human participants or animals performed by any of the authors.
